# A Micro-RNA Connection in **BRaf** ^**V600E**^-Mediated Premature Senescence of Human Melanocytes

**DOI:** 10.1155/2012/913242

**Published:** 2012-04-24

**Authors:** Gang Ren, Jingwei Feng, Ila Datar, Aaron H. Yeung, Srinivas Vinod Saladi, Yongqing Feng, Ivana de la Serna, Kam C. Yeung

**Affiliations:** Department of Biochemistry and Cancer Biology, University of Toledo College of Medicine, Health Science Campus, Toledo, OH 43614-2598, USA

## Abstract

Recent high-throughput-sequencing of the cancer genome has identified oncogenic mutations in *BRaf* genetic locus as one of the critical events in melanomagenesis. In normal cells, the activity of *BRaf* is tightly regulated. Gain-of-function mutations like those identified in melanoma frequently lead to enhanced cell-survival and unrestrained growth. The activating mutation of *BRaf* will also induce the cells to senesce. However, the mechanism by which the oncogenic *BRaf* induces the senescent barrier remains poorly defined. microRNAs have regulatory functions toward the expression of genes that are important in carcinogenesis. Here we show that expression of several microRNAs is altered when the oncogenic version of *BRaf* is introduced in cultured primary melanocytes and these cells undergo premature cellular senescence. These include eight microRNAs whose expression rates are significantly stimulated and three that are repressed. While most of the induced microRNAs have documented negative effects on cell cycle progression, one of the repressed microRNAs has proven oncogenic functions. Ectopic expression of some of these induced microRNAs increased the expression of senescence markers and induced growth arrest and senescence in primary melanocytes. Taken together, our results suggest that the change in microRNA expression rates may play a vital role in senescence induced by the oncogenic *BRaf*.

## 1. Introduction

Unregulated oncogene expression during cancer development causes cancer cells to senesce prematurely, a gene-directed program that irrevocably induces cell cycle arrest [1–4]. First described in cell culture [5], oncogene-induced senescence (OIS) has been confirmed *in vivo* as a vital mechanism that constrains the malignant progression of many tumors [3]. Unregulated oncoproteins promote senescence by activating effector pathways that are cell-type and oncogene specific. Recent progress identified different regulatory circuitries of OIS, but much remains to be learned [6]. High-throughput sequencing of the cancer genomes has identified *BRaf* kinase as the most frequently mutated (50–70%) oncogene in melanoma [7]. About 90% of BRaf gain-of-function mutations are at position 600 with glutamic acid (E) inserted for valine (V) [7]. BRaf is a serine/threonine protein kinase that functions directly downstream of the small GTPase Ras and upstream of the MEK and ERK mitogen-activated protein kinase (MAPK) cascade. This mutation significantly increases BRaf kinase activity toward MEK, causing constitutive BRaf-MEK-ERK signaling [7, 8]. The *BRaf *gain-of-function Val to Glu mutation is required for cell viability, anchorage-independent growth, and proliferation of BRaf^V600E^-positive melanoma cell lines in both cell-based studies and *in vivo *mouse models [9–11]. Furthermore, a Phase I clinical trial with a potent inhibitor of BRaf^V600E^ kinase demonstrates that *BRaf*-mutant melanomas are highly dependent on BRaf kinase activity [12]. Besides its frequent presence in melanoma, *BRaf* ^V600E^ mutations are also present in up to 82% of melanocytic nevi with classical hallmarks of senescence [13]. Importantly, sustained BRaf^V600E^ expression in human melanocytes induces premature senescence [13]. The gain-of-function mutation in *BRaf* may be the cause of growth arrest in nevi. Indeed, in studies with genetically modified mouse models, the activated *BRaf* ^V600E^ allele plays a causal role in promoting benign melanocytic hyperplasia *in vivo* [14, 15]. However the underlying mechanisms driving B-Raf^V600E^-mediated senescence in melanocytes remain undefined.

MicroRNAs are small noncoding RNA species of 20–22 nucleotides that have critical functions across a myriad of biological processes [16–19]. Aberrant expression of several microRNAs has also been reported in melanocytic nevi [20]. In view of the emerging important role of microRNAs in tumorigenesis and cell-cycle arrest, microRNAs may play a causal role in BRaf^V600E^-induced senescence in melanocytic nevi. BRaf^V600E^-induced senescence requires the activation of a transcription program with multiple components [21–23]. The mechanism that integrates the diverse components into a coordinated response to the BRaf^V600E^ mutation remains undefined. Each microRNA targets ~200 mRNA molecules [18]. Because of their pleiotropic potentials, microRNAs are attractive candidates as master regulators of the premature senescence transcription program. In this study, by ectopically expressing BRaf^V600E^ in primary melanocytes, we identified 8 different microRNAs whose expressions were correlated with the BRaf gain-of-function activity. Importantly, two of the identified microRNAs, when expressed individually, are sufficient to induce premature senescence in primary melanocytes in the absence of *BRaf* mutation.

## 2. Results and Discussion

**Figure 1:**
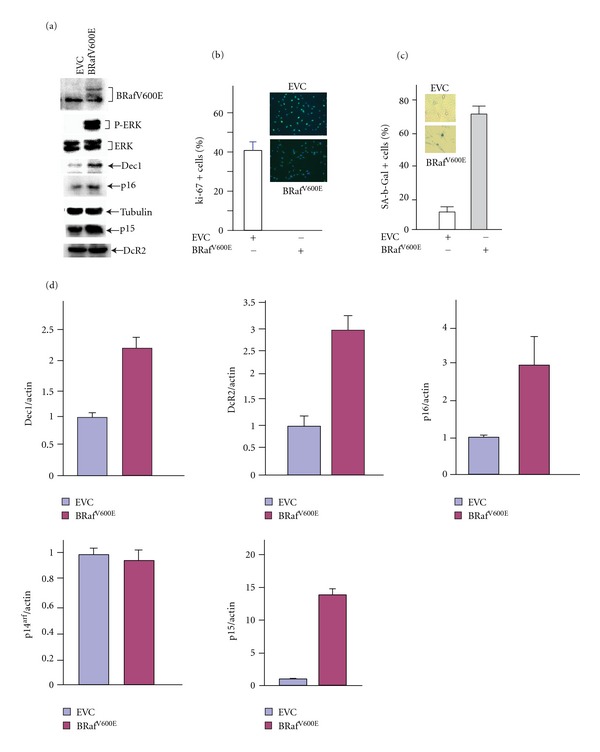
Melanocytes were infected with control retroviral empty vector (EVC) or with a vector expressing BRaf^V600E^. Four days after infection, cells were harvested for (a) immunoblotting with specific Abs as indicated or (d) total RNA. The expression levels of the indicated senescence markers in BRaf^V600E^ expressing or control cells were quantified by qRT-PCR and normalized to the level of b-actin. The values represent the means and standard deviation from three independent experiments. Cells were also isolated 4 days after infection, fixed, and stained for (b) ki-67 or (c) SA-*β*-Gal activity. Insets: representative fields are shown for control EVC and B-Raf^V600E^ expressing cells stained with ki-67 Ab or positive for SA-*β*-Gal activity.

**Figure 2:**
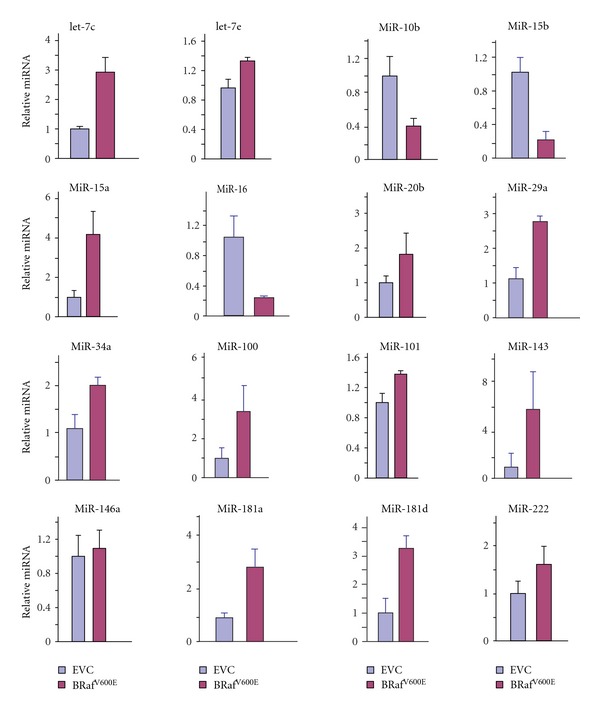
Melanocytes were infected with EVC or with vector expressing BRaf^V600E^. Cells were harvested 4 days after infection and the expression levels of the indicated miRNAs were quantified by qRT-PCR with specific primers (Biosciences) and normalized to the level of U6 RNA. The values represent the means and standard deviation from three independent experiments.

To identify putative microRNAs involved in OIS, we detected microRNAs induced by *BRaf* ^V600E^ in melanocytes using a focused PCR-based microarray system that enabled assessment of 52 microRNAs (SABiosciences). As expected, expression of BRaf^V600E^ in primary human melanocytes induced constitutively high levels of ERK1/2 phosphorylation (Figure 1(a)) promoting growth arrest (Figure 1(b)) and senescence as detected by the expression of senescence-associated *β*-galactosidase (SA-*β*-Gal) (Figure 1(c)), Dec1, p16, p15, and DcR2 (Figures 1(a) and 1(d)). At a time point prior to the detection of senescence, we collected total RNA and enriched for small RNAs as recommended by the manufacturer. By enriching for small RNAs, this system is designed to detect mature microRNA expression levels. The levels of each microRNA were quantified relative to the levels of four control small RNAs. Among the 52 microRNAs that we examined, 13 were significantly induced and three (miR-10b, -15b, and -16) were greatly repressed by *BRaf* ^V600E^ in primary melanocytes (data not shown). Subsequently, quantitative real-time PCR (qRT-PCR) was used to further examine the effect of *BRaf* ^V600E^ on the expression of these microRNAs with specific primers for each individual microRNA. Eight out of the 13 that were stimulated in the original screen were consistently induced more than 2-fold by *BRaf* ^V600E^ (Figure 2). Based on the documented expression of these microRNAs in clinical melanocytic nevi, we selected four of them for further study [20, 24, 25].

**Figure 3:**
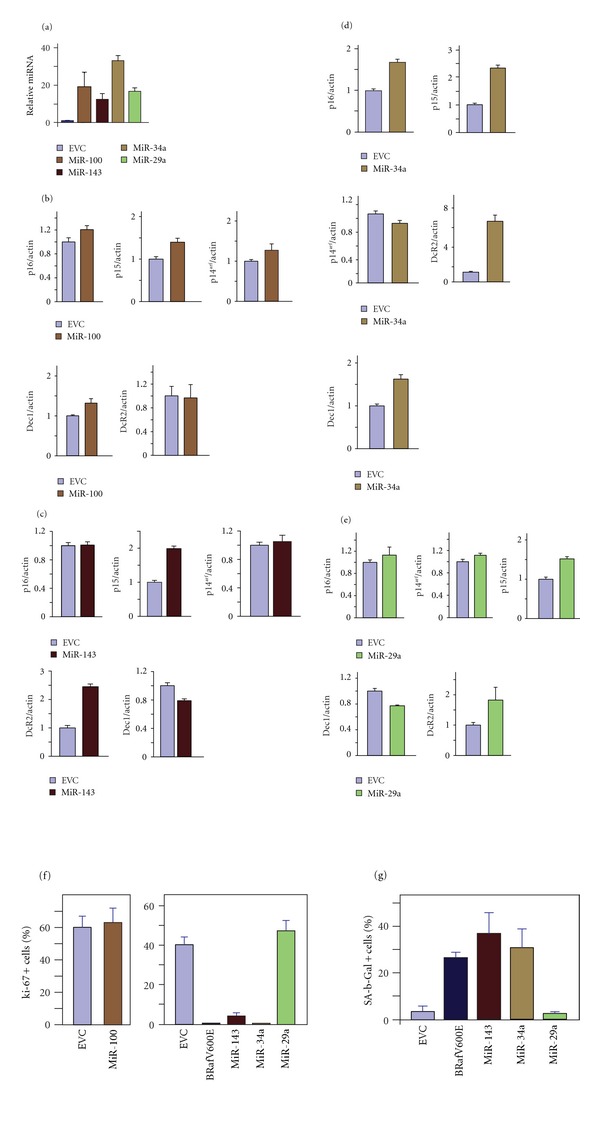
Melanocytes were infected with EVC or with vector expressing the indicated miRNA. (a) Quantification of miRNAs expression by qRT-PCR in indicated miRNA expressing cells. (b)–(e) The expression levels of the indicated senescence markers in the indicated miRNA expressing cells were quantified by qRT-PCR and normalized to the level of b-actin. The values represent the means and standard deviation from three independent experiments. Cells infected with the indicated miRNA expressing virus were also isolated after selection, fixed, and stained for (f) ki-67 or (g) SA-*β*-Gal activity.

To examine their causal role in cellular senescence in melanocytes, we expressed individual microRNAs in primary human skin melanocytes by retroviral transduction. The expression of microRNAs was confirmed by qRT-PCR with specific primers (Figure 3(a)). The effects of individual microRNA expression on the expression of five senescence markers were determined by qRT-PCR (Figures 3(b)–3(e)). Three out of the four microRNAs tested had an observable effect on the expression of at least one of the senescence markers. These are microRNAs 143, 34a, and 29a. While expression of miR-34a increased *p*16^INK4a^, *p*15^INK4b^ *, DcR2,* and *Dec1* transcripts, expression of miR-143, or 29a increased the *p*15^INK4b^ and* DcR2 *transcripts (Figures 3(c)–3(e)). To investigate directly their role in cellular senescence, we measured cell proliferation by quantifying the number of melanocytes expressing the proliferation marker protein ki-67 due to the expression of miR-143, 34a, or 29a. Expression of miR-143 or -34a but not -29a or -100 led to a decrease in melanocyte proliferation as measured by the expression of proliferation marker protein ki-67. Importantly, the decreased expression of ki-67 correlated with intense activity of SA-*β*-Gal (Figure 3(f)).

Oncogenic *BRaf* induced premature senescence in melanocytes with a concomitant increase in the expression of senescence markers p16^INK4a^, p15^INK4b^, Dec1, and DcR2. In addition, we showed that oncogenic *BRaf* also significantly increased the expression of miR-143, miR-34a, let-7c, miR-15a, miR-29a, miR-100, miR-181a, and miR-181d. By specific Watson-Crick base pairing between the seed region of microRNA and sites within the mRNA 3′UTR, each microRNA can potentially target different groups of mRNAs for regulation. Expression of miR-143, -34a, or -29a led to the increased expression of some of the senescence markers. It is therefore possible that a combined expression of the identified microRNAs is responsible for the observed upregulation of senescence markers in oncogenic *BRaf* expressing melanocytes.

Among the microRNAs that are induced by the *BRaf* gain-of-function mutation, only miR-143 or -34a is sufficient to induce growth arrest and senescence when ectopically expressed in primary melanocytes. miR-34a negatively regulates cell cycle G1/S transition genes cyclin D1, cyclin E2, CDK4, and CDK6 and causes G1 arrest [26–28] when expressed in human primary diploid fibroblasts. In human cancer cells, miR-143 is one of the most notable tumor suppressor miR-RNAs, which can directly inhibit oncogene KRAS translation and block the downstream signal pathways [29]. Restoring miR-143 expression inhibited proliferation and induced apoptosis by targeting BCL2 [30, 31]. In agreement with these studies, we showed that miR-34a or -143 induced premature senescence in primary human melanocytes. Ectopic expression of miR-34a increased expression of p16^INK4a^, p15^INK4b^, Dec1, and DcR2. Unlike miR-34a, miR-143 only increased expression of p15^INK4b^ and DcR2. Since both miR-34a and -143 expression caused senescence, it can be inferred that expression of p16^INK4a^ or Dec1 is not essential for oncogenic BRaf-mediated premature senescence. Concordantly, the presence of p16^INK4a^ was also previously found not to be required for senescence induced by gain-of-function *BRaf* mutation in melanocytes [13]. It is presently not clear which mRNAs, miR-34a or -143, target for repression in melanocytes, nor do we know how oncogenic *BRaf *induces their expression.

By targeting different subset of genes for regulation, microRNAs can function either as oncogenes or tumor suppressor genes. As expected, all the identified induced miR-RNAs have tumor suppressive functions. For instance, miR-29, which has three family members, negatively impinges on cancer cell survival by directly targeting the mRNAs of the regulatory subunit of *PI3 kinase* (p85a) and *CDC42* for regulation. miR-29a could also directly decrease *CDK6* expression and cause G1/S growth arrest in [32]. Consistent with its tumor suppressive role, miR-29 expression is frequently downregulated in multiple cancers. However despite its proven role in cell cycle regulation, expression of miR-29a is not sufficient to induce senescence in melanocytes. It is possible that miR-29a is required but not sufficient for oncogenic *BRaf*-induced senescence. It is also possible that the effect of miR-29a on cell cycle progression is cell-context dependent.

We reason that if microRNA is part of the mechanism that integrates the diverse components into a coordinated response to the *BRaf* ^V600E^ mutation, we should also observe downregulation of oncogenic microRNAs. Indeed, miR-10b, which increases the expression of oncogene RhoC by directly targeting its transcriptional repressor HOXD10 [33], is consistently repressed in melanocytes harboring the *BRaf* ^V600E^ mutation. In addition to miR-10b, we also observed expression levels of miR-15b and -16 downregulated. miR-15/16 belongs to a very unique group of microRNAs. miR-16 has 2 transcripts—one is called miR-16-1 located on Chromosome (Chr) 13 and shares the same transcript with miR-15a forming the miR-15a/16 cluster. The other miR-16 is named miR-16-2, which together with miR-15b is located at Chr 3 as the miR-15b/16 cluster. Interestingly while miR-15b and 16 were downregulated, the miR-15a was upregulated in melanocytes expressing *BRaf* ^V600E^. Since our miR-16 primer detected both miR-16-1 and -16-2 transcripts, it is unknown at present which miR-16 was downregulated by oncogenic *BRaf* expression. The miR-15/16 family is known to have a negative effect on cell proliferation by targeting mRNAs for various cell-growth-associated genes [34–36]. It is therefore not clear why the downregulation of miR-15b and -16 is favored in melanocytes, which are undergoing senescence. Alternatively it is also possible that miR-5b/16 has unidentified oncogene targets.

Although it is clear that *BRaf* induces senescence in melanocytes by activating MEK and ERK, the downstream effectors of its cytostatic effect remain to be identified [13, 37]. *BRaf* ^V600E^-mediated senescence also requires additional effector pathways involving several immune and growth mediators such as IL6, IL8, and IGFBP7 [6]. However, the mechanism that integrates the diverse pathways into a coordinate response to oncogenic stimulation has not been defined. In this study, we identified several microRNAs whose expression rates were stimulated by oncogenic *BRaf*. Expression of four of these microRNAs induced the expression senescence-surrogate markers. Among them, miR-143 or -34a alone can induce growth arrest and senescence when expressed in primary melanocytes.

Melanoma is a cancer of melanocytes and is the most deadly form of skin cancer. The transformation of melanocytes to malignant melanoma is a stepwise process fueled by the accumulation of mutations in critical growth and survival regulatory genes. The development of benign nevi from melanocytes is the first phenotypic change that can be detected during the progression of normal melanocytes to malignant melanoma. Studies with mouse models have demonstrated the causal role of the activated *BRaf* ^V600E^ allele in promoting benign melanocytic hyperplasia [14, 15]. The underlying mechanisms that drive the transformation of melanocytes to a precancerous nevi are poorly understood. Since microRNAs have multiple regulatory targets [18], our results together with the expression of these microRNAs in clinical melanocytic nevi [20] strongly suggest that the identified microRNAs may play a vital coordinative role in *BRaf-*induced senescence in melanocytic nevi. It is conceivable that benign nevi transform into melanoma of uncontrolled growth following subsequent mutations that inactivate the oncogenic B-Raf-mediated senescence pathways. As such a thorough understanding of how oncogenic *BRaf* induces senescence will allow us to elucidate the molecular mechanisms that drive the conversion of melanocytes to preneoplastic nevi and transition to melanoma.

## 3. Materials and Methods

### 3.1. Cell Lines and Reagents

Human melanocytes were from Cascade Biologics (Portland, OR, USA) or Yale Cell Culture Core Facility. Human melanocytes were grown in Media 254 with added growth supplements (Cascade Biologics).

### 3.2. Plasmid Constructs

Retroviral expression vectors (miR-vec) for miR-100, miR-15a/16-1, and -181a were a kind gift of Reuven Agami. The miRNA minigenes -29a, -34a, -143, -181d, -15b, and -15b/16-2 were PCR amplifided from genomic human DNA, cloned downstream of the CMV promoter in miR-vec, and sequence verified. The primers for the miRNA minigenes cloning were the following:

miR-29a Forward: 5′-gcGGATCCCTGGAACCAATCCCTCAA
miR-29a Reverse: 5′-gcGAATTCGCTCCTTTCCCATCATCT
miR-34a Forward: 5′-gcGGATCCGGCTGGTCTTGAACTCCT
miR-34a Reverse: 5′-gcGAATTCCACTGGCTACTATTCTCCCTA
miR-143 Forward: 5′-gcGGATCCTCAAGGTTTGGTCCTGGGTG
miR-143 Reverse: 5′-gcGAATTCCGTGAAGCAGATCGTGGC
miR-15b Forward: gcggatccAAGGGGATGATTATGAAG
miR-15b Reverse: gcgaattcAGTGGAACAAGTATGTCAGT
miR-15b/16 Forward: gcggatccGACTTGGACCATAATAGA
miR-15b/16 Reverse: gcgaattcTAGGTGCTTAGGTAAATC
miR-181d Forward: gcggatccGACCGTTGAGTGGACCC
miR-181d Reverse: gcgaattcTCCAGCCAGAGCCCATCC

### 3.3. Isolation of Genomic DNA from Primary Melanocytes

One 10 cm cultured plate of subconfluent primary melanocytes was harvested by trypsin digestion. Trypsin was subsequently neutralized and cells were collected by centrifugation and washed with PBS. The washed cell-pellet was resuspended with 1.2 mL cell resuspension buffer (10 mM Tris-HCL, 10 mM NaCl, 1.5 mM MgCl_2_) by gently pipetting up and down. Cells were digested with 8 mL of sucrose/proteinase K lysis buffer (27% sucrose, 1xSSC, 1 mM EDTA, 1%SDS, 200 ug/mL proteinase K) overnight at 37°C. The next day digested cells were extracted with 10 mL phenol: chloroform: isoamyl alcohol (25 : 24 : 1) and the DNA was precipitated with 1 volume isopropanol. DNA was dissolved and extracted with phenol/chloroform/isoamyl alcohol again followed by precipitation with 1/3 volume of 7.5 M ammonium acetate (pH 7.4). Precipitated DNA was washed with 70% ethanol and dissolved in TE (pH 7.4).

### 3.4. Analysis of SA-b-Galactosidase Activity

The senescence-associated b-galactosidase activity in culture cell was detected with a staining kit purchased from Cell Signaling Technology according to the manufacturer's specifications.

### 3.5. Ki-67 Staining

Cells were plated on laminin-coated cover slips (Laminin, L 2020, SIGMA). After 48 hours, cells were washed with PBS and fixed with 100% cold Methanol for 10 minutes. Fixed cells were then incubated in blocking solution (0.1% BSA in PBS) for 30 minutes. Primary antibodies, 200–300 *μ*L (1 : 100 in block solution), specific for ki-67 (sc-15402, Santa Cruz) were added onto each cover slips and incubated overnight at 4°C. Primary antibody-stained cells were washed 3 times for 3 minutes each with 0.1% BSA-PBS blocking solution. Washed cells were subsequently incubated with FITC-conjugated secondary antibody (1 : 200 diluted in blocking solution) for 1 hour at room temperature followed by one wash with blocking solution for 3 minutes, two PBS washes for 3 minutes each, and two washes with dH_2_O. The nuclei of ki-67 stained cells were visualized with DAPI (4 *μ*g/mL in dH_2_O) staining for 10 min. Cells were rinsed with dH_2_O twice before mounting onto glass slides with DAKO fluorescent mounting medium (Dako, s3032). The glass slides were dried in the hood for 5–10 minutes. The cover slip edges were sealed with nail enamel polisher and were dried in the hood for an addition 10–20 min.

### 3.6. Antibodies

The BRaf polyclonal antibodies were from Upstate (number 07-453). The Phospho-p44/42 MAPK (Thr202/Tyr204) (E10) Mouse mAb (number 9106) and p44/42 MAP Kinase Antibodies (number 9102) were from Cell Signaling. Dec1 (s-8) (sc-101023), p16 (JC8) (sc-56330), and p15 (C-20) (sc-612) antibodies were from Santa Cruz. DcR2 Polyclonal Antibody (AAP-371) was from Assay designs. Monoclonal anti-*α* Tubulin Clone B-5-1-2 (T-5168) was from SIGMA.

### 3.7. Quantitative Real-Time RT-PCR

Total cellular RNA was extracted with the Trizol reagent (Invitrogen) and reverse transcribed using random hexamer primers (Applied Biosystems). The resulting cDNAs were used for PCR using SYBR-Green Master PCR mix (QIAGEN) in triplicates. PCR and data collection were performed on ABI7500 (Applied Biosystems). *β*-actin, GAPDH, and HPRT were used as an internal standard. The gene-specific primers were as follows:

human p14
  R: 5′-CAT GAC CTG GTC TTC TAG GAA GC-3′
  F: 5′-CCC TCG TGC TGA TGC TAC TGA-3′
human p15
  R: 5′-GCA TGC CCT TGT TCT CCT CG-3′
  F: 5′-GGG AAA GAA GGG AAG AGT GTC GTT-3′
human p16
  R: 5′-GGT TGT GGC GGG GGC AGT T-3′
  F: 5′-GGG GGC ACC AGA GGC AGT-3′
human DcR2
  R: 5′-CCG GGG GAT GGT GGC AGA GT-3′
  F: 5′-CGC TCG AGC AGG GCG CTA TC-3′
human Dec1
  R: 5′-CGA TGA GCC GGT GCG GCA AT-3′
  F: 5′-CCG GGA CTG GAG CAC GGA GA-3′
b-actin
  F: 5′-ATCTGGCACCAGACCTTCTACAATGAGCTGCG-3′
  R: 5′-CGTCATACTCCTGCTTGCTGATCCACATCTGC-3′
glyceraldehyde-3-phosphate dehydrogenase
  F: 5′-TGCACCACCAACTGCTTAGC-3′
  R: 5′-GGCATGGACTGTGGTCATGAG-3′

### 3.8. Real-Time PCR to Check Expression Levels of miRNAs

cDNA from the obtained miRNAs was synthesized using RT^2^ miRNA first strand kit (SABiosciences). Amount of miRNA used was 200 ng. The cDNA was then amplified using miRNA-specific primer. RNU6, SNORD 44, and SNORD 48 were used as controls. Real-time PCR results were analyzed using the ddCT method.

### 3.9. Isolation of miRNA

Small RNA from primary melanocytes was prepared using RNeasy Mini kit protocol (Qiagen). Cells were harvested for RNA using TRIzol (Invitrogen). Chloroform was added to the tubes containing homogenate (20% of the volume of TRIzol used). After shaking the tubes vigorously, the tubes were incubated at RT for 2-3 minutes and centrifuged (12,000× g) for 15 mins at 4°C. The upper aqueous layer was transferred to new tubes and 1 volume of 70% ethanol was added. The sample was mixed and applied to the RNeasy minispin column and centrifuged at 8000× g (10,000 rpm) for 15 s at RT. The larger RNAs are bound to the column filter. The flow-through contained miRNAs and was further purified according to the manufacturer's specifications (Qiagen).
